# Human umbilical cord mesenchymal stem cells improve disease characterization of Sjogren's syndrome in NOD mice through regulation of gut microbiota and Treg/Th17 cellular immunity

**DOI:** 10.1002/iid3.1139

**Published:** 2024-01-10

**Authors:** Yao Zou, Wei Xiao, Dongzhou Liu, Xianyao Li, Lihua Li, Lijuan Peng, Ying Xiong, Haina Gan, Xiang Ren

**Affiliations:** ^1^ Jinan University Guangzhou Guangdong China; ^2^ Department of Rheumatology and Immunology, Changde Hospital, Xiangya School of Medicine Central South University Changde Hunan China; ^3^ Department of Rheumatology and Immunology Shenzhen People's Hospital Shenzhen Guangdong China

**Keywords:** gut microbiota, human umbilical cord mesenchymal stem cells, Sjogren's syndrome, Treg/Th17

## Abstract

**Background:**

For the unclear pathogenesis of Sjogren's syndrome (SS), further exploration is necessary. Mesenchymal stem cells (MSCs) and derived exosomes (MSCs‐exo) have exhibited promising results in treating SS.

**Object:**

This study aimed to investigate the effect and mechanism of human umbilical cord MSCs (UC‐MSCs) on SS.

**Methods:**

Nonobese Diabetic (NOD) mouse splenic T cells were co‐cultured with UC‐MSCs and UC‐MSCs‐exo, and interferon‐gamma (IFN‐γ), interleukin (IL)‐6, IL‐10, prostaglandin E2 (PGE2), and transforming growth factor‐β1 (TGF‐β1) levels in the supernatant were assessed by quantitative real‐time polymerase chain reaction and enzyme‐linked immunosorbent assay. Co‐cultured T cells were injected into NOD mice via the tail vein. The inflammatory cell infiltration in the intestine and the submandibular gland was characterized by hematoxylin‐eosin staining. Treg/Th17 homeostasis within the spleen was determined by flow cytometry. Gut microbiota was detected by 16S rRNA sequencing, and the relationship between differential microbiota and Treg/Th17 cytokines was analyzed by the Pearson correlation coefficient.

**Results:**

UC‐MSCs, UC‐MSCs‐exo, and NOD mouse splenic T cells were successfully cultured and identified. After T cells were co‐cultured with UC‐MSCs and UC‐MSCs‐exo, both IFN‐γ and IL‐6 were decreased while IL‐10, PGE2, and TGF‐β1 were increased in transcriptional and translational levels. UC‐MSCs and UC‐MSCs‐exo partially restored salivary secretion function, reduced Ro/SSA antibody and α‐Fodrin immunoglobulin A levels, reduced inflammatory cell infiltration in the intestine and submandibular gland, raised proportion of Treg cells, decreased IFN‐γ, IL‐6, IL‐2, IL‐17, lipopolysaccharide, and tumor necrosis factor‐alpha levels, and raised IL‐10, Foxp3, and TGF‐β1 levels by affecting co‐cultured T cells. The intervention of UC‐MSCs and UC‐MSCs‐exo improved intestinal homeostasis in NOD mice by increasing microbiota diversity and richness. Additionally, differential microbiota was significantly associated with Treg/Th17 cytokine levels.

**Conclusion:**

Human UC‐MSCs and UC‐MSCs‐exo improved disease characterization of SS in NOD mice through regulation of gut microbiota and Treg/Th17 cellular immunity.

## Graphical abstract

The intervention of umbilical cord (UC)‐mesenchymal stem cells (MSCs) and UC‐MSC and derived exosomes improved intestinal homeostasis in NOD mice by increasing microbiota diversity and richness. Additionally, differential microbiota was significantly associated with Treg/Th17 cytokine levels.

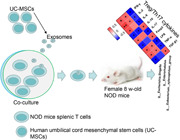

## 1 INTRODUCTION

Sjögren's syndrome (SS) is a chronic autoimmune disease characterized by inflammation and damage of the exocrine glands, especially the salivary and lacrimal glands.^1^ Systemic manifestations such as fatigue, dry skin, and joint and muscle pain occur in 30%–40% of patients with primary SS (pSS), and lymphoma occurs in 2%–5% of them.^2^, ^3^ Although significant progress has been made in elucidating the pathophysiological mechanisms of SS, there is still no cure for this disease. Currently, SS treatment primarily focuses on alternative and symptomatic therapies to relieve the disease symptoms of patients.^4^ Therefore, the search for a safe treatment that not only can regulate immune disorders to relieve the disease symptoms of SS but also repair damaged tissue is the goal pursued by the rheumatology community.

The study has revealed that the severity of ocular and systemic diseases in SS is negatively correlated with the diversity of gut microbiota.^5^ The gut microbiota of pSS patients showed a raised abundance of pro‐inflammatory microbiota and a reduced abundance of anti‐inflammatory microbiota. Among pro‐inflammatory microbiota, *Escherichia‐Shigella* is a major factor associated with the dysregulation of gut microbiota, which may promote intestinal damage and influence amino acid metabolism.^6^ Further studies on the gut microbiota of pSS patients showed that the relative abundance of conditionally pathogenic bacteria was high. However, the relative abundance of beneficial or symbiotic butyrate‐producing bacteria was low. These changes may impair intestinal barrier function and thus promote the chronic inflammatory process associated with pSS by increasing the secretion of pro‐inflammatory cytokines such as interleukin (IL)−6, IL‐12, IL‐17, and tumor necrosis factor‐alpha (TNF‐α), and downregulating forkhead box protein 3 (Foxp3) level that is involved in the development and function of regulatory T cells (Tregs).^7^ In animal model studies, 11‐week‐old Nonobese Diabetic (NOD) mice spontaneously exhibited symptoms associated with SS, along with depressive‐like behavior and abnormal gut microbiota composition.^8^ Functional studies of germ‐free mice colonized with human gut microbiota from SS patients and healthy controls found that gut microbiota modulated the ocular surface health of SS‐humanized mice by affecting CD4^+^Foxp3^+^ Tregs development in the ophthalmic draining lymph nodes.^9^ It has been found that the decrease in the absolute number of Treg cells, the main lymphocyte mediating immune tolerance, may be one of the main pathogenesis of SS, but further reasons remain to be clarified.^10^ Therefore, we boldly speculate that treating SS by improving gut microbiota imbalance and restoring cellular immune function may be a safe and effective strategy.

Human umbilical cord mesenchymal stem cells (UC‐MSCs) are known to induce CD4^+^Foxp3^+^ T cells in NOD mice and humans in vitro and effectively interfere with an autoimmune attack during SS by inducing a nonresponsive T cell state in vivo and upregulation of Tregs.^11^ In addition, UC‐MSCs could relieve SS‐associated lung inflammation.^12^ Studies have demonstrated that MSCs from different sources could inhibit the secretion of pro‐inflammatory cytokines, reduce inflammatory cell infiltration in salivary glands and lacrimal glands, and restore exocrine gland function of NOD mice.^13^, ^14^ However, as cell therapy, intravenous injection of MSCs may be at risk of pulmonary capillary interception, ectopic osteoblastoma, and induction of an alloimmune response. In addition, the biological distribution effect of MSCs is affected by their characteristics, microenvironment, immunity, inflammation, and cancer, so their clinical application in SS treatment is limited.^15^

Studies have shown that exosomes contain messenger RNA (mRNA), microRNA, proteins, lipids, and other intercellular message transducers, which regulate the survival and function of immune cells. Due to high bioavailability, convenient use, and low immunogenicity, the application of exosomes in disease treatment has a broad prospect.^16^ MSCs‐exo could imitate the immune regulation of MSCs while avoiding the risks of MSCs treatment. Research has shown that UC‐MSCs and derived exosomes (UC‐MSCs‐exo) could regulate CD4^+^ T cell proliferation and early apoptosis, inhibit T helper 17 (Th17) cell differentiation, and promote Treg cell differentiation to restore Th17/Treg balance in pSS patients.^17^ Another study has suggested that intervention with MSCs derived from labial glands and their exosomes alleviates inflammatory cell infiltration in salivary glands and partially recovers salivary secretion function in NOD mice. Moreover, the secretion of IL‐17, interferon‐gamma (IFN‐γ), and IL‐6 by T cells was decreased while that of transforming growth factor‐β1 (TGF‐β1) and IL‐10 increased.^18^ Therefore, MSCs‐derived exosomes are a potential novel treatment strategy for SS as cell‐free therapy.

In this study, UC‐MSCs‐exo were co‐cultured with NOD mouse splenic T cells in vitro. The co‐cultured T cells were injected back into NOD mice by tail vein for in vivo intervention. This study aims to explore the possibility of UC‐MSCs in SS treatment by regulating gut microbiota disturbance, intestinal injury, inflammatory cell infiltration in the submandibular gland, and immune cell differentiation.

## 2 MATERIALS AND METHODS

### 2.1 Isolation, culture, and identification of human UC‐MSCs

Fresh umbilical cords were obtained from informed, consenting mothers at the First People's Hospital of Changde City. Under sterile conditions, after washing with phosphate‐buffered saline (PBS) and removing blood vessels, umbilical cords were cut into small segments and evenly laid on culture plates. Then, the special medium for MSCs (AW‐MC025; Abiowell) was added to culture plates and placed in an incubator (DH‐160I; SANTN) with 5% CO_2_ at 37°C. The special medium consisted of basal medium, 1% growth supplement, 5% fetal bovine serum (FBS), and 1% Penicillin/Streptomycin. The medium was changed every 3 days. When confluence reached 70%–80%, the cells were digested using trypsin (AWC0232; Abiowell) to harvest primary cells. The cells were passaged at a ratio of 1:3. The third‐generation human UC‐MSCs were used in the experiment. The morphology of UC‐MSCs was observed by the inverted biological microscope (DSZ2000X; Cnmicro). The surface antigen phenotypes of UC‐MSCs, including CD34, CD44, CD45, CD73, CD90, CD105, and human leukocyte antigen‐DR isotype (HLA‐DR), were identified by flow cytometry. The adipogenic and osteogenic differentiation of UC‐MSCs was assessed by Oil red O staining and Alizarin Red staining, respectively.^19^

### 2.2 Extraction and identification of UC‐MSCs‐exo

UC‐MSCs‐exo was extracted using ExoQuick‐TC PLUS™ exosomes extraction kit (#WQPL10TC‐1; SBI) and differential centrifugal method. The morphology of UC‐MSCs‐exo was characterized by transmission electron microscope (HT7700; Hitachi High‐technologies) at 100 kV. Photos were taken with a charge‐coupled device camera (MORADA G3, EMSIS GMBH) followed by quantification with Image J software (National Institutes of Health). The expression levels of CD9 (1: 2000, 60232‐1‐Ig; Proteintech), CD63 (1: 500, 25682‐1‐AP; Proteintech), tumor susceptibility gene 101 (TSG101, 1: 5000, 28283‐1‐AP; Proteintech), Calnexin (1: 10,000, 10427‐2‐AP; Proteintech) in UC‐MSCs‐exo were detected by Western blot with β‐actin used as the reference protein.

### 2.3 Extraction of NOD mice splenic T cells

NOD mice were killed by cervical dislocation, and the spleen was taken and placed on the 40 μm cell strainer that was premoistened with 2 mL complete medium, cut with ophthalmic scissors, and ground with the base of the piston of a 5 mL syringe. The cell strainer was rinsed with 10 mL of Roswell Park Memorial Institute (RPMI)‐1640 medium. The above rinse solution was centrifuged for 5 min to obtain cell precipitation. Subsequently, 5 mL erythrocyte lysate was added, cleaved at room temperature for 3 min, and centrifuged for 5 min. The cells were resuspended with 1 mL buffer and 1.8 × 10^7^ cells were counted. NOD mouse splenic T cells were cultured in RPMI‐1640 medium (AW‐MC002; Abiowell) supplemented with 10 FBS and 1% Penicillin/Streptomycin under the condition of 5% CO_2_ at 37°C.

### 2.4 Immunomagnetic bead sorting of CD4^+^ T cells

A naive CD4^+^ T Cell isolation kit (130‐104‐453; Miltenyi) was used for cell sorting. NOD mouse splenic T cells were resuspended in 80 μL of the pre‐cooled buffer. Next, 20 μL of biotin‐antibody was added and incubated for 5 min. Then, 30 μL of precooled buffer, 20 μL of antibiotin microbeads, and 10 µL of CD44 microbeads were added before incubation in the refrigerator for 5 min. The large size (LS) separation columns were placed on a magnetic rack, and the above cells were added to the LS separation columns. The columns were washed twice with buffer, and the collected cells that passed through were used for subsequent experiments (approximately 6.1 × 10^6^ cells were counted after sorting). The purity of CD4^+^ T cells and the ratio of Tregs to Th17 cells were analyzed by flow cytometry.

### 2.5 Co‐culture of NOD mouse splenic T cells with UC‐MSCs and UC‐MSCs‐exo

UC‐MSCs (5 × 10^4^/mL) were co‐cultured with splenic T cells (5 × 10^5^/mL) from NOD mice for 48 h.^20^ UC‐MSCs‐exo (200 μg) was co‐cultured with splenic T cells (5 × 10^5^/mL) from NOD mice for 48 h.

### 2.6 Animal grouping and intervention

NOD mice are commonly used as spontaneous SS model mice.^21^, ^22^, ^23^ Female 8‐week‐old NOD mice were obtained from Beijing HFK Bio‐Technology Co. Ltd. The housing conditions involve maintaining the temperature at 25 ± 1°C and the humidity at 45%–55%, with a 12 h light/dark cycle. The NOD mice have access to food and water and are acclimated for 1 week. NOD mice were randomly divided into five groups, including Model, UC‐MSCs, UC‐MSCs‐exo, UC‐MSCs+T cell, and UC‐MSCs‐exo+T cell groups, with six mice in each group. The intervention was performed in NOD mice by caudal intravenous injection. NOD mice in the UC‐MSCs group were injected with 1 × 10^6^ human UC‐MSCs.^11^ NOD mice in the UC‐MSCs‐exo group were injected with 200 μg UC‐MSCs‐exo.^24^ NOD mice in the UC‐MSCs+T cell group were injected with 1 × 10^6^ T cells co‐cultured with UC‐MSCs. NOD mice in the UC‐MSCs‐exo+T cell group were injected with 1 × 10^6^ T cells cocultured with UC‐MSCs‐exo. NOD mice in the Model group were treated with the same dose of normal saline. After continuous intervention for 8 weeks, mice were killed, and feces, intestines, peripheral blood, and submandibular glands were taken for analysis.

### 2.7 Enzyme‐linked immunosorbent assay (ELISA)

The whole blood was placed at room temperature for 2 h followed by centrifugation at 1000*g* at 4°C for 15 min, and the supernatant was analyzed. The cell cultures were centrifuged at 4°C 1000*g* for 15 min to collect the supernatant. The levels of IFN‐γ (KE10001; Proteintech), IL‐2 (CSB‐E04627m; CUSABIO), IL‐6 (KE10007; Proteintech), IL‐10 (CSB‐E04594m; CUSABIO), IL‐17 (KE10020; Proteintech), prostaglandin E2 (PGE2, YJ028719; MLBIO), transforming growth factor‐beta1 (TGF‐β1, KE10005; Proteintech), SS‐related antigen A (Ro/SSA) antibody (69‐20401; MSKBIO), α‐Fodrin immunoglobulin A (IgA) (ml001942; MLBIO), Foxp3 (YJ037859; MLBIO), lipopolysaccharide (LPS, CSB‐E13066m; CUSABIO), and TNF‐α (CSB‐E04741m; CUSABIO) were determined by ELISA kits.

### 2.8 Quantitative real‐time polymerase chain reaction (RT‐qPCR)

The total RNA in CD4^+^ T cells was extracted by using the Trizol reagent (15596026; Thermo). mRNA was reverse‐transcribed into complementary DNA following the guidance of the mRNA reverse transcription kit (CW2569; CWBIO), followed by RT‐qPCR with UltraSYBR Mixture (CW2601; CWBIO). The primers used here were shown in Table 1. The expression level of mRNA was calculated by the $2^{‐∆∆C_{t}}$ method with β‐actin as the internal reference.

**Table 1:** Sequences of the primers.

Gene name	Forward (5′−3′)	Reverse (5′−3′)
β‐actin	ACCCTGAAGTACCCCATCGAG	AGCACAGCCTGGATAGCAAC
IFN‐γ	TGAATGTCCAACGCAAAGCAA	TTACTGGGATGCTCTTCGACCT
IL‐6	GCAATAACCACCCCTGACCCAA	GCTACATTTGCCGAAGAGCC
IL‐10	ACCTGCCTAACATGCTTCGAGA	CTCAGCTTGGGGCATCACCT
PGE2	CGCCGAGATCCAGATGGTCA	ACGAATACTCGCACCACGAG
TGF‐β1	AGCAACAATTCCTGGCGATACCTC	CAATTTCCCCTCCACGGCTCA

Abbreviations: IFN‐γ, interferon‐gamma; IL, nterleukin; PGE2, prostaglandin E2; TGF‐β1, transforming growth factor‐beta1.

### 2.9 Saliva flow test

NOD mice were anesthetized with tribromoethanol (0.36 g/kg, A18706; Thermo Fisher Scientific) and subcutaneously injected with pilocarpine as a saliva stimulant (0.5 mg/kg, P6503; Sigma). According to the literature description, saliva was collected on a cotton swab for 20 min every 2 weeks.^25^ The swab was weighed before and after the collection. Saliva weight was converted to volume, namely, 1 μg represented 1 μL.

### 2.10 Hematoxylin‐eosin (HE) staining

The submandibular gland and intestine from NOD mice were fixed with 4% paraformaldehyde, embedded in paraffin, and cut into 2 μm sections for HE staining analysis. The slices were dewaxed to water, and stained with hematoxylin (AWI0001a; Abiowell) followed with eosin (AWI0029a; Abiowell). The slices were placed in xylene for 10 min for transparency and then sealed with neutral gum (AWI0238a; Abiowell) for observation by microscope.

### 2.11 16S rRNA sequencing

Rectal feces were collected from five groups of NOD mice with six samples in each group. DNA was extracted by TIANamp Stool DNA Kit (#DP328‐02; Tiangen) from fecal samples. PCR amplification and library construction were conducted by Phusion enzyme (K1031; APExBIO) and the V3‐V4 region primers (341F 5′‐CCTACGGGNGGCWGCAG‐3′ and 805R 5′‐GACTACHVGGGTATCTAATCC‐3′) of the 16S rRNA gene. An Illumina NovaSeq. 6000 instrument\s was used for paired‐end (PE250) sequencing to collect raw data. Qiime 2 (2020.2) was used to conduct data quality control, calculate the α diversity index, and measure relative abundance. Each amplicon sequence variant/operating taxonomic units (ASV/OTU) sequence was annotated by referring to the SilvA‐132‐99 database, and the corresponding species information and abundance distribution were obtained. R software (Venn Diagram package) and the Jvenn web page (http://www.bioinformatics.com.cn/static/others/jvenn/example.html) were used to analyze common and unique ASVs in groups. Linear discriminant analysis effect size analysis (LefSe, https://github.com/SegataLab/lefse) was applied to evaluate the differential microbiota.

### 2.12 Statistical analysis

Statistical analysis was achieved by GraphPad Prism 8.0 (GraphPad Software Inc.). Data were shown as mean ± standard deviation (SD). The normal distribution and homogeneity of variance of data were analyzed by the Kolmogorov–Smirnov test and exploratory descriptive statistics test. Comparisons between multiple groups were analyzed by one‐way analysis of variance (ANOVA) and Tukey′s post‐hoc test. Comparisons between groups at different time points were analyzed by two‐way ANOVA with Bonferroni as a post hoc test. The correlation between differential microbiota and Treg/Th17 cytokines was analyzed by the Pearson correlation coefficient. The difference was statistically significant when *p* < .05.

## 3 RESULTS

### 3.1 Identification of UC‐MSCs, UC‐MSCs‐exo, and NOD mouse splenic T cells

**Figure 1:**
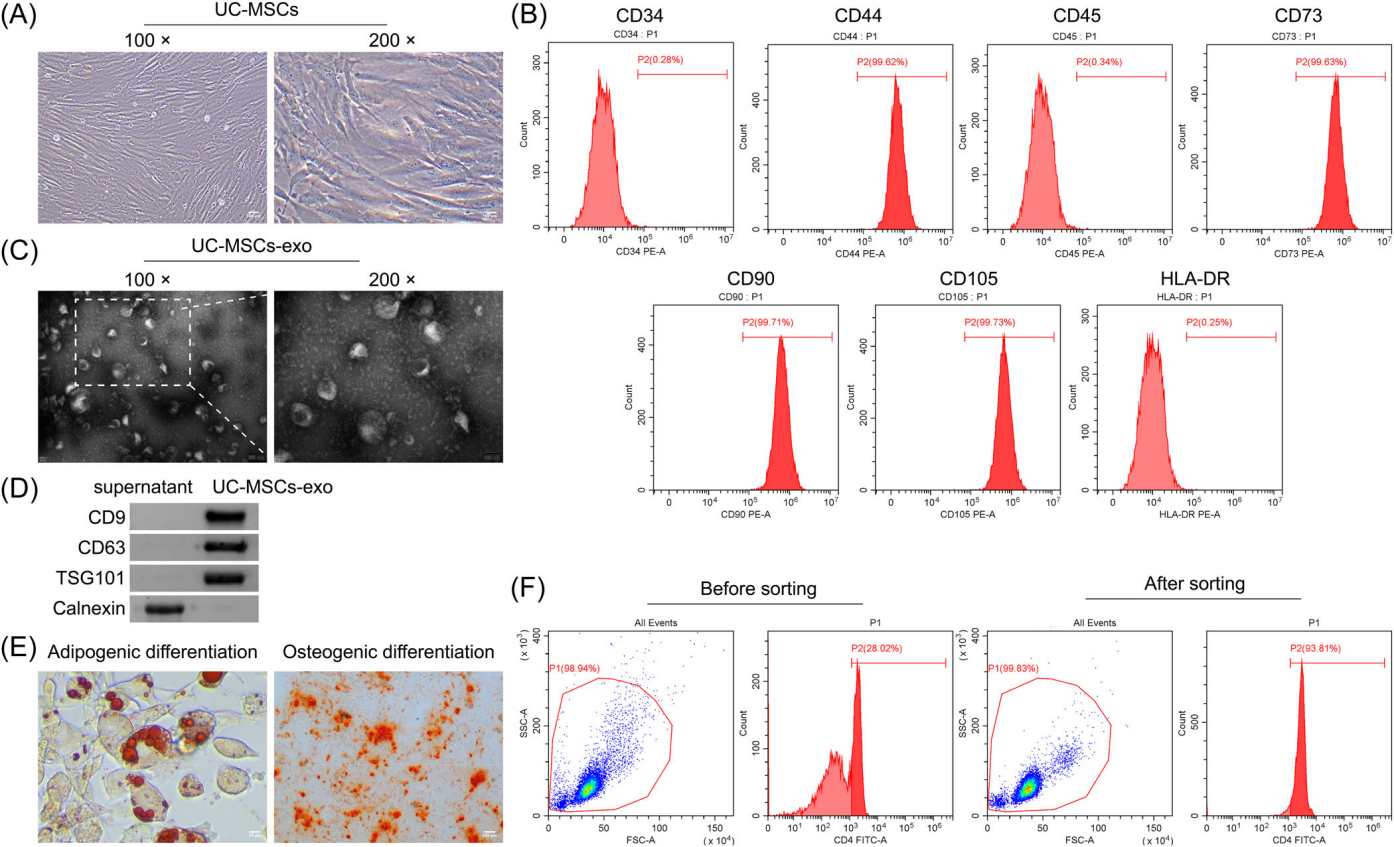
Identification of umbilical cord mesenchymal stem cells (UC‐MSCs), UC‐MSCs and derived exosomes (UC‐MSCs‐exo), and Nonobese Diabetic (NOD) mouse splenic T cells. (A) The morphology of UC‐MSCs was characterized by an optical microscope. Scale bar: 100 μm (left), 50 μm (right). (B) The expressions of the surface antigens (CD34, CD44, CD45, CD73, CD90, CD105, and human leukocyte antigen‐DR isotype) of the third‐generation UC‐MSCs were determined by flow cytometry. (C) The morphology of UC‐MSCs‐exo was characterized by transmission electron microscope. Scale bar: 200 nm (left), 100 nm (right). (D) The expressions of CD9, CD63, TSG101, and Calnexin in UC‐MSCs‐exo were assessed by Western blot. (E) Adipogenic (left) and osteogenic (right) differentiation of UC‐MSCs were assessed by Oil red O staining and Alizarin Red staining, respectively. Scale bar: 25 μm (left), 100 μm (right). (F) The splenic CD4^+^ T cells of NOD mice were selected by immunomagnetic beads.

First, we characterized the morphology of UC‐MSCs and noted that they exhibited a uniformly long spindle‐shaped appearance and grew in a typical vortex‐like arrangement (Figure 1A). Further, flow cytometry analysis of the third‐generation UC‐MSCs revealed highly expressed biomarkers including CD44, CD73, CD90, and CD105, along with low expression of CD34, CD45, and HLA‐DR (Figure 1B). Interestingly, the appearance of UC‐MSCs‐exo was found to be typically saucer‐shaped or hemispherical with a concave side, with a diameter of approximately 100 nm (Figure 1C). Western blot analysis of the biomarker proteins of exosomes showed that CD9, CD63, and TSG101 were highly expressed in UC‐MSCs‐exo (Figure 1D). After lipogenic induction, the shape of UC‐MSCs gradually presented an oval shape, and the cytoplasm was filled with red oil droplets. After osteogenic induction, the shape of UC‐MSCs gradually changed from spindle‐shaped to polygonal, with numerous bright red calcium nodules visible (Figure 1E). Moreover, CD4^+^ T cells were sorted by immunomagnetic beads, with a positive rate being 93.81% (Figure 1F). The above proved that UC‐MSCs, UC‐MSCs‐exo, and NOD mouse splenic T cells were successfully identified and cultured.

### 3.2 UC‐MSCs and UC‐MSCs‐exo affected IFN‐γ, IL‐6, IL‐10, PGE2, and TGF‐β1 levels in co‐cultured T cells

**Figure 2:**
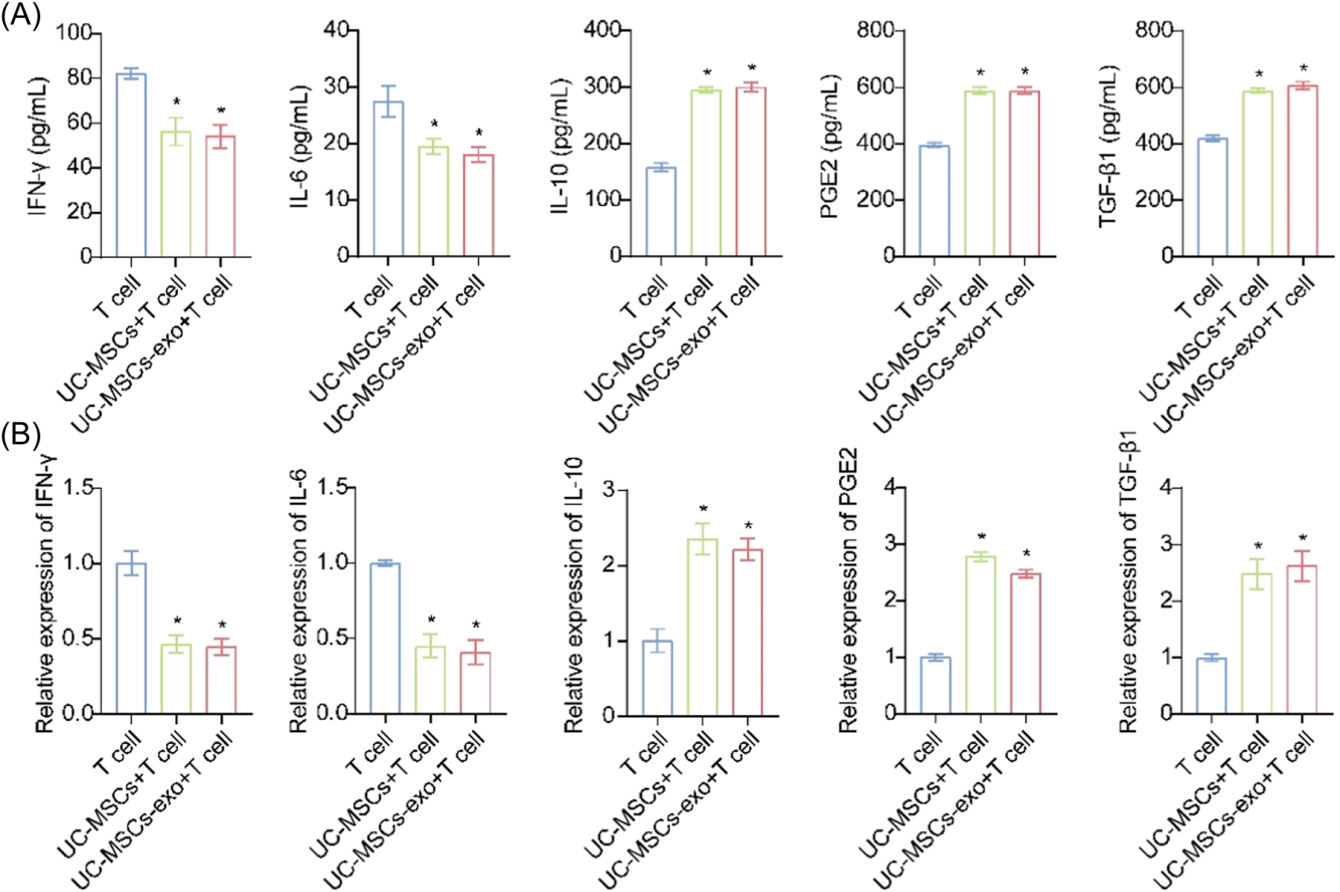
Umbilical cord mesenchymal stem cells (UC‐MSCs) and UC‐MSCs and derived exosomes (UC‐MSCs‐exo) affected interferon‐gamma (IFN‐γ), interleukin (IL)‐6, IL‐10, prostaglandin E2 (PGE2), and transforming growth factor‐beta1 (TGF‐β1) levels in cocultured T cells. (A) Levels of IFN‐γ, IL‐6, IL‐10, PGE2, and TGF‐β1 were measured by enzyme‐linked immunosorbent assay. (B) The mRNA expressions of IFN‐γ, IL‐6, IL‐10, PGE2, and TGF‐β1 were quantified by Quantitative real‐time polymerase chain reaction. * *p* < .05 versus T cell.

To explore the impact of UC‐MSCs and UC‐MSCs‐exo on T cells, ELISA and RT‐qPCR were performed to analyze the changes in component expression of T cells after co‐culture. Upon co‐culture of T cells with UC‐MSCs and UC‐MSCs‐exo, the contents of IFN‐γ and IL‐6 in the supernatant were decreased, while those of IL‐10, PGE2, and TGF‐β1 were increased (Figure 2A). Correspondingly, when T cells were co‐cultured with UC‐MSCs and UC‐MSCs‐exo, the mRNA expressions of IFN‐γ and IL‐6 were downregulated, and those of IL‐10, PGE2, and TGF‐β1 were upregulated (Figure 2B). These results suggested that UC‐MSCs and UC‐MSCs‐exo affected IFN‐γ, IL‐6, IL‐10, PGE2, and TGF‐β1 levels in co‐cultured T cells.

### 3.3 UC‐MSCs and UC‐MSCs‐exo improved disease characterization of SS in NOD mice by affecting co‐cultured T cells

**Figure 3:**
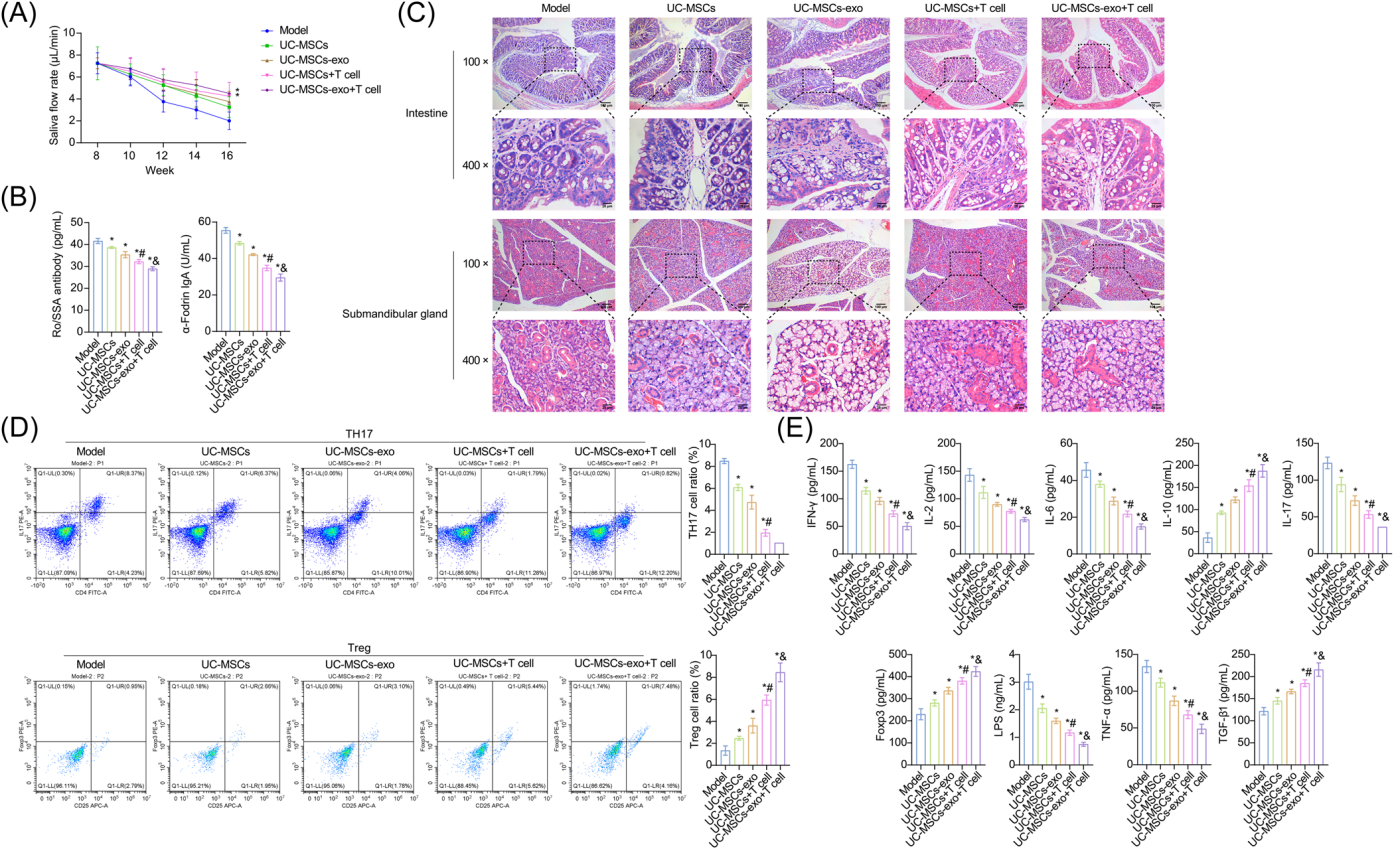
Umbilical cord mesenchymal stem cells (UC‐MSCs) and UC‐MSCs and derived exosomes (UC‐MSCs‐exo) improved disease characterization of Sjogren's syndrome (SS) in Nonobese Diabetic (NOD) mice by affecting co‐cultured T cells. (A) Saliva flow. (B) The levels of Ro/SSA antibody and α‐Fodrin immunoglobulin A were detected by enzyme‐linked immunosorbent assay (ELISA). (C) Pathological changes of the submandibular gland and intestine were observed by hematoxylin‐eosin staining. Scale bar: 100 μm (up), 25 μm (down). (D) The ratio of Treg/Th17 cells in the spleen was determined by flow cytometry. (E) The levels of interferon‐gamma (IFN‐γ), interleukin (IL)‐2, IL‐6, IL‐10, IL‐17, Foxp3, lipopolysaccharide, tumor necrosis factor‐alpha, and transforming growth factor‐β1 were analyzed by ELISA. *p < .05 versus Model, ^#^ *p* < .05 versus UC‐MSCs, and *p* < .05 versus UC‐MSCs‐exo.

To explain the impact of UC‐MSCs and UC‐MSCs‐exo on SS, T cells were co‐cultured with these cells and subsequently injected into NOD mice via the tail vein. The saliva flow of NOD mice in UC‐MSCs+T cell and UC‐MSCs‐exo+T cell groups was increased compared to the Model group (Figure 3A). Additionally, injection of T cells co‐cultured with UC‐MSCs and UC‐MSCs‐exo led to a significant reduction in Ro/SSA antibody and α‐Fodrin IgA levels in the serum of NOD mice (Figure 3B). To investigate whether UC‐MSCs and UC‐MSCs‐exo affect the submandibular glands and intestines of NOD mice, we performed histopathological analysis. The results demonstrated that co‐culture of T cells with UC‐MSCs and UC‐MSCs‐exo alleviated damage to the submandibular gland and intestinal in NOD mice, reduced inflammatory cell infiltration, and improved glandular arrangement (Figure 3C). Moreover, co‐culture of T cells with UC‐MSCs and UC‐MSCs‐exo raised the ratio of Treg cells and reduced that of Th17 cells in the spleen of NOD mice (Figure 3D). Next, we performed ELISA to detect levels of Treg/Th17 cell cytokines in the serum of NOD mice. Co‐culture of T cells with UC‐MSCs and UC‐MSCs‐exo raised Foxp3, IL‐10, and TGF‐β1 levels while decreasing IFN‐γ, IL‐6, IL‐2, IL‐17, LPS, and TNF‐α levels (Figure 3E). These results revealed that UC‐MSCs and UC‐MSCs‐exo improved disease characterization of SS in NOD mice by affecting co‐cultured T cells.

### 3.4 UC‐MSCs and UC‐MSCs‐exo improved gut microbiota imbalance of SS in NOD mice by affecting co‐cultured T cells

**Figure 4:**
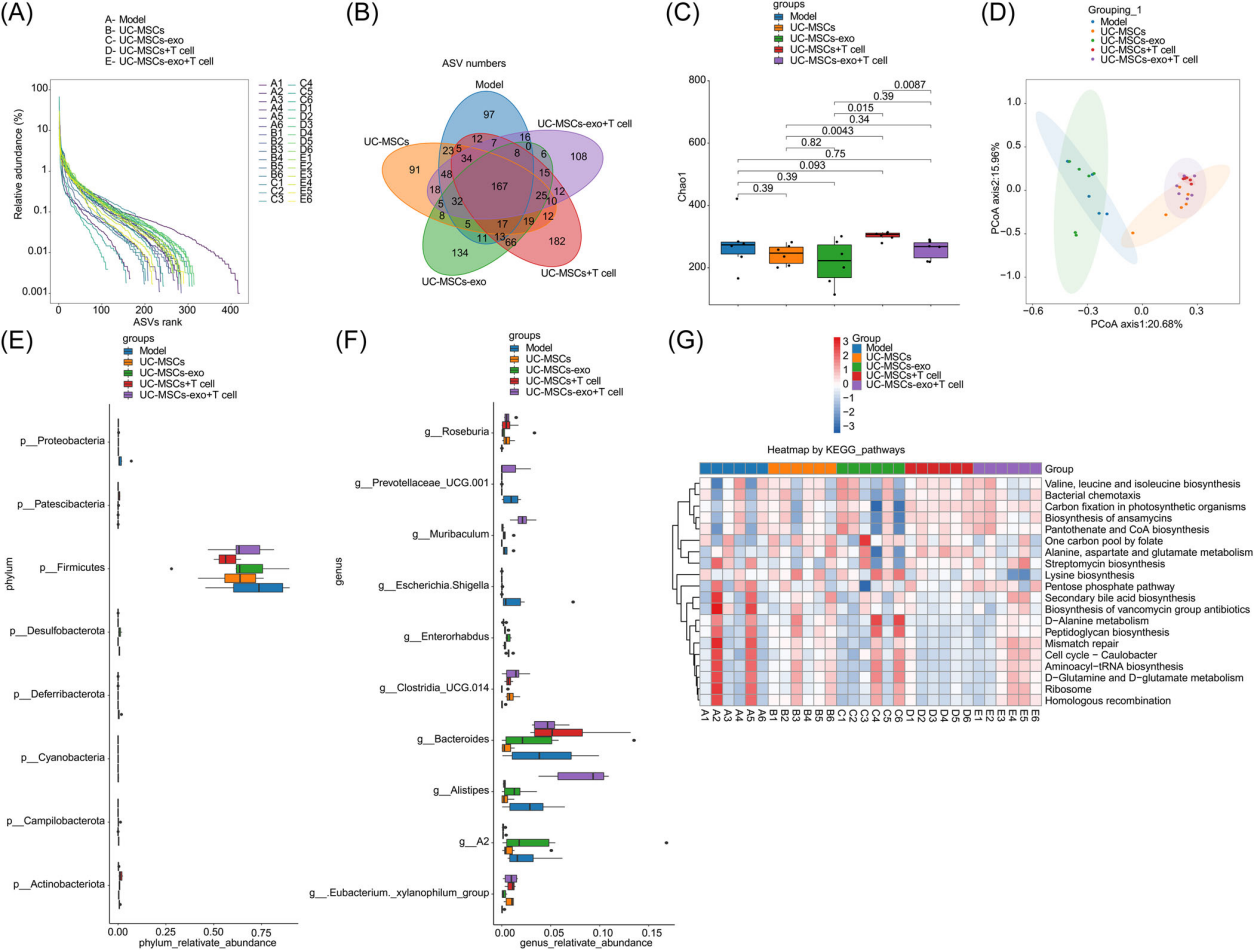
Umbilical cord mesenchymal stem cells (UC‐MSCs) and UC‐MSCs and derived exosomes (UC‐MSCs‐exo) improved gut microbiota imbalance of Sjogren's syndrome in Nonobese Diabetic mice by affecting co‐cultured T cells. (A) Rank‐abundance curve. (B) Venn diagram. (C) α diversity analysis. (D) β diversity analysis. (E, F) Differential microbiota analysis at phylum and genus levels. (G) Heatmap for the predicted function of differential microbiota using Kyoto Encyclopedia of Genes and Genomes.

To investigate the impact of UC‐MSCs and UC‐MSCs‐exo on the gut microbiota of NOD mice, rectal feces from each group were subjected to 16S rRNA sequencing. The rank‐abundance curve exhibited that the richness and evenness of gut microbiota in the Model group were low and that in the UC‐MSCs+T cell and UC‐MSCs‐exo+T cell groups were high (Figure 4A). Veen diagram displayed that co‐culture of T cells with UC‐MSCs and UC‐MSCs‐exo raised gut microbiota number in NOD mice (Figure 4B). Compared with the Model group, the α diversity index of UC‐MSCs+T cell and UC‐MSCs‐exo+T cell groups was increased, but the change was not significant (Figure 4C). The principal component analysis exhibited overlapping between the Model group and UC‐MSCs‐exo group as well as among the UC‐MSCs, UC‐MSCs+T cell, and UC‐MSCs‐exo+T cell groups. The first principal component accounted for 20.68% of the variance, while the second principal component accounted for 15.96% (Figure 4D). At the phylum level, differential microbiota included *Proteobacteria*, *Patescibacteria*, *Firmicutes*, *Desulfobacterota*, *Deferribacterota*, *Cyanobacteria*, *Campilobacterota*, and *Actinobacteriota* (Figure 4E). At the genus level, differential microbiota included *Roseburia*, *Prevotellaceae_UCG.001*, *Muribaculum*, *Escherichia‐Shigella*, *Enterorhabdus*, *Clostridia_UCG.014*, *Bacteroides*, *Alistipes*, *A2*, and *Eubacterium._xylanophilum_group* (Figure 4F). Furthermore, the heatmap indicated certain similarities and differences in the pathways enriched from the Kyoto Encyclopedia of Genes and Genomes among the five groups (Figure 1G). These findings proved that UC‐MSCs and UC‐MSCs‐exo improved gut microbiota imbalance of SS in NOD mice by affecting co‐cultured T cells.

### 3.5 Correlation between differential microbiota and Treg/Th17 cytokines

**Figure 5:**
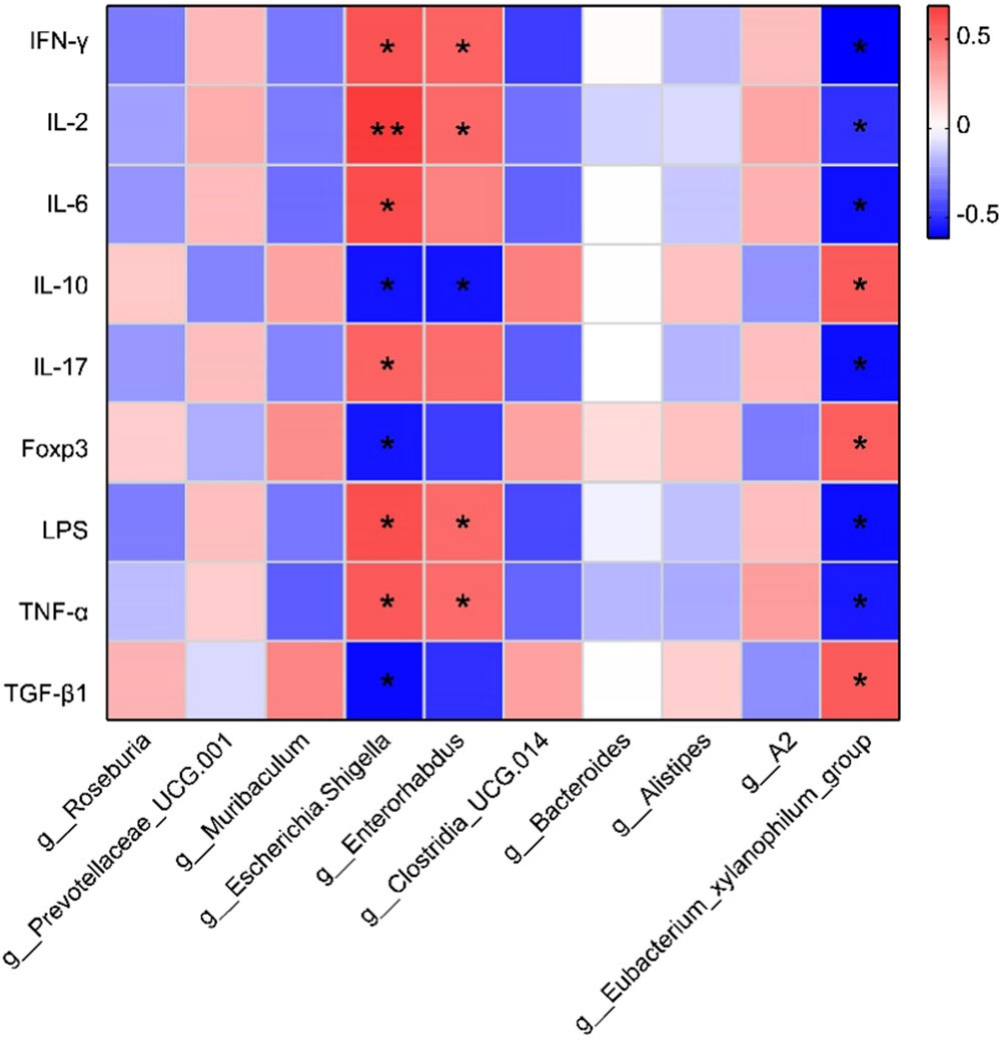
Heatmap of the relationship of differential microbiota with Treg/Th17 cytokines. *p < .05, **p < .01. IFN‐γ, interferon‐gamma; IL, interleukin; TGF‐β1, transforming growth factor‐beta1; TNF‐α, tumor necrosis factor‐alpha.

To illustrate the relationship between gut microbiota and Treg/Th17 cellular immunity, we conducted a Pearson correlation coefficient analysis on the differential microbiota and Treg/Th17 cytokines. The heatmap displayed a significant positive correlation between the abundance of *Escherichia‐Shigella* and IFN‐γ, IL‐2, IL‐6, IL‐17, LPS, and TNF‐α levels, along with a significant negative correlation with IL‐10, Foxp3, and TGF‐β1 levels. Additionally, we observed a significant positive correlation between *Enterorhabdus* and IFN‐γ, IL‐2, LPS, and TNF‐α levels, whereas a significant negative correlation with the level of IL‐10. The *Eubacterium_xylanophilum_group* showed a significant positive correlation with IL‐10, Foxp3, and TGF‐β1 levels, but a significant negative correlation with IFN‐γ, IL‐2, IL‐6, IL‐17, LPS, and TNF‐α levels. These findings illustrated that differential microbiota has an impact on Treg/Th17 cellular immunity (Figure 5).

## 4 DISCUSSION

SS is a systemic autoimmune disease with a complex mechanism that still requires further exploration. The application of MSCs in SS treatment has been reported, but their potential mechanisms of action vary among studies.^26^ In this study, we selected the ideal SS model, female NOD mice, and verified the possibility of using UC‐MSCs or UC‐MSCs‐exo to restore the gut microbiota, reduce inflammatory cell infiltration in the gut and submandibular glands, and regulate the Treg/Th17 balance for SS treatment through tail vein injection of T cells co‐cultured with UC‐MSCs or UC‐MSCs‐exo.

MSCs possess characteristics such as immunosuppressive function and nonimmunogenicity, making them potentially useful for the treatment of various autoimmune diseases.^27^ Ro/SSA autoantibodies are widely accepted and considered to be diagnostic tools for classification in the context of SS.^28^ In addition, α‐Fodrin antibodies can be detected in the serum of most pSS patients.^29^ Treatment with UC‐MSCs in 24 pSS patients resulted in a decrease in α‐Fodrin and Ro/SSA antibodies after 1 month.^30^ Other studies have also indicated a reduction in Ro/SSA antibodies after injection of MSCs and MSCs‐exo in mice.^31^ In this study, injection of T cells co‐cultured with UC‐MSCs or UC‐MSCs‐exo into NOD mice resulted in a reduction in the concentration of Ro/SSA and α‐Fodrin antibodies in the serum. NOD mice are characterized by reduced saliva flow and increased lymphocyte infiltration in the salivary glands, but transplantation of MSCs can restore saliva function and reduce lymphocyte infiltration.^32^ In this study, UC‐MSCs and UC‐MSCs‐exo restored saliva secretion function and reduced inflammatory cell infiltration in the submandibular gland, as well as repairing intestinal damage by influencing T cells in NOD mice.

Treg/Th17 balance is essential in the pathogenesis of autoimmune diseases and the body's defense response.^33^ The ratio of Th17 cells and the level of IL‐17 in the peripheral blood of pSS patients are significantly elevated compared to healthy controls, which jointly promote the onset of pSS.^34^ In IL‐17 knockout SS mouse models, glandular pathological damage is significantly reduced, and saliva and tear gland secretion function, as well as saliva flow rate, are significantly improved.^35^ Other studies have shown that similar to IL‐17, IFN‐γ is also a key cytokine in the development of SS salivary gland inflammation.^36^ In addition, TNF‐α can maintain and enhance the influx of neutrophils into inflammatory sites, exerting a synergistic pro‐inflammatory effect.^37^ In mice, TGF‐β and IL‐6 initiate the differentiation of Th17 cells, and in the absence of IL‐6, TGF‐β, and IL‐21 can also induce the differentiation of Th17 cells, but the role of IL‐6 is stronger.^38^ LPS is an endotoxin that can cause inflammatory diseases and disrupt intestinal barrier integrity.^39^ IL‐10 is a cell factor that inhibits inflammatory response, and in the serum of pSS patients, IL‐10 levels are significantly decreased, which is due to the inhibition of Treg cells in the diseased state, leading to a decrease in IL‐10 secretion.^40^ Studies have shown that Foxp3 is a genetic marker of Treg cells, and its decreased expression is directly related to SS glandular inflammation.^41^ Other studies have found that short‐term low‐dose IL‐2 therapy can restore Th17/Treg balance in the peripheral blood of patients with pSS.^42^ It has also been reported that Treg cells are reduced in the peripheral blood of pSS patients.^43^ In this study, after co‐culturing T cells with UC‐MSCs or UC‐MSCs‐exo, and injecting them into NOD mice, the proportion of Treg cells in the spleen increased, while the proportion of Th17 cells decreased. Additionally, Foxp3, IL‐10, and TGF‐β1 levels in the peripheral blood increased, while IFN‐γ, IL‐6, IL‐2, IL‐17, LPS, and TNF‐α levels decreased. These results indicate that UC‐MSCs or UC‐MSCs‐exo alleviate SS by regulating the Treg/Th17 balance and promoting the release of anti‐inflammatory cytokines.

Studies have shown that the dysregulation of gut microbiota homeostasis may contribute to SS pathogenesis. Significant changes in the diversity, composition, and function of gut microbiota have happened in SS.^44^ The gut microbiota of untreated pSS patients has low richness and evenness.^45^ In this study, the gut microbiota of NOD mice was disrupted, with a decrease in both α and β diversity. However, after intervention with T cells co‐cultured with either UC‐MSCs or UC‐MSCs‐exo, the gut microbiota disorder was improved, manifesting as increased diversity and the abundance of specific microorganisms. Among the gut microbiota, *Firmicutes* and *Bacteroidetes* are two major bacteria, and the *Firmicutes/Bacteroidetes* ratio has a significant impact on maintaining gut homeostasis. An imbalanced ratio may lead to various diseases, such as inflammation, autoimmune diseases, and cancer.^46^ In this study, the gut microbiota of NOD mice was highly enriched in *Firmicutes*, while the abundance of *Bacteroidetes* was decreased. UC‐MSCs and UC‐MSCs‐exo improved the imbalance of the *Firmicutes/Bacteroidetes* ratio by influencing the co‐cultured T cells. Additionally, the functionality of different microbiota also underwent certain changes. Therefore, it could be seen that UC‐MSCs and UC‐MSCs‐exo exerted their effects by regulating the structure and function of the gut microbiota in NOD mice by influencing the co‐cultured T cells.

Previous studies have observed the depletion of anti‐inflammatory microbiota producing butyric acid and the enrichment of pro‐inflammatory microbiota in the systemic analysis of gut microbiota changes in a range of rheumatic diseases.^47^ *Escherichia‐Shigella* is associated with inflammation, and its abundance is positively correlated with pro‐inflammatory cytokine levels.^48^ *Enterorhabdus* promotes LPS‐induced intestinal inflammation and dysbiosis in mice.^39^ *Eubacterium._xylanophilum_group* is named as the main contributor of anti‐inflammatory metabolites such as short‐chain fatty acids.^49^ Here, the relative abundance of *Escherichia‐Shigella* and *Enterorhabdus* in the gut microbiota of NOD mice injected with T cells co‐cultured with UC‐MSCs or UC‐MSCs‐exo was reduced, while the relative abundance of *Eubacterium._xylanophilum_group* was increased. Pearson correlation coefficient analysis revealed that *Escherichia‐Shigella* and *Enterorhabdus* were positively correlated with IFN‐γ, IL‐2, IL‐6, IL‐17, LPS, and TNF‐α levels, while negatively correlated with IL‐10, Foxp3, and TGF‐β1 levels. However, *Eubacterium._xylanophilum_group* showed opposite correlations. These results suggested that gut microbiota was associated with cytokine release and subsequently improved the inflammatory state. UC‐MSCs and UC‐MSCs‐exo could promote the enrichment of beneficial gut microbiota by affecting co‐cultured T cells, thereby improving intestinal inflammation and other disease characterizations of SS.

## 5 CONCLUSION

In summary, both UC‐MSCs and UC‐MSCs‐exo could modulate Treg/Th17 balance, restore gut microbiota homeostasis, and improve the disease characterization of SS. This study will enrich insights into the pathogenesis of SS and the exploration of therapeutic approaches.

## AUTHOR CONTRIBUTIONS

**Yao Zou**: Conceptualization; data curation; formal analysis; writing—original draft. **Wei Xiao**: Data curation; formal analysis; visualization; writing—review and editing. **Dongzhou Liu**: Data curation; formal analysis; visualization; writing—review and editing. **Xianyao Li**: Data curation; formal analysis; visualization; writing—review and editing. **Lihua Li**: Data curation; formal analysis; writing—review and editing. **Lijuan Peng**: Data curation; formal analysis; writing—review and editing. **Ying Xiong**: Data curation; formal analysis; writing—review and editing. **Haina Gan**: Data curation; formal analysis; writing—review and editing. **Xiang Ren**: Conceptualization; validation; writing—review and editing.

## CONFLICT OF INTEREST STATEMENT

The authors declare no conflict of interest.

## ETHICS STATEMENT

All animal experiments were approved by Animal Ethics Committee of Changde Hospital, Xiangya School of Medicine, Central South University (approval number 2021‐050‐01) and humane care was given to experimental animals in strict accordance with the Reduction, RepIacement, and Refinement (3R) principle.

## Data Availability

All original data can be obtained from corresponding authors when necessary.
